# Supplementary material for: Matching variants for functional characterization of genetic variants
Source: G3 (Bethesda). 2023 Nov 2;13(12):jkad227. doi: 10.1093/g3journal/jkad227 (PMC10700107; doi:10.1093/g3journal/jkad227)
Supplement: jkad227_Supplementary_Data [file jkad227_supplementary_data.zip › Supplementary_Figure_1_G3-2023-404469.pdf]

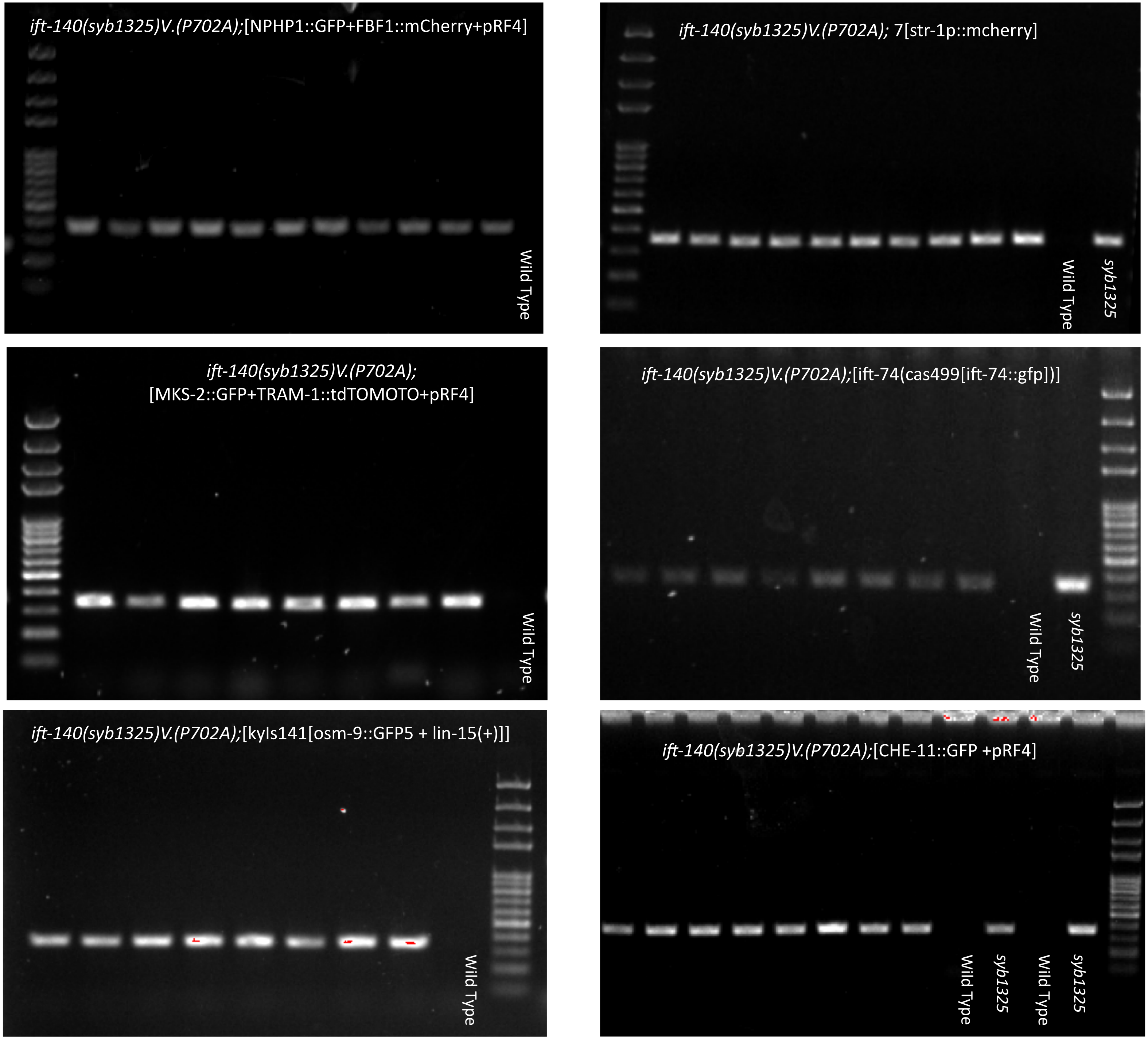
ift-140(syb1325)V.(P702A);[NPHP1::GFP+FBF1::mCherry+pRF4]
Wild Type
ift-140(syb1325)V.(P702A); 7[str-1p::mcherry]
Wild Type
syb1325
ift-140(syb1325)V.(P702A);
[MKS-2::GFP+TRAM-1::tdTOMOTO+pRF4]
Wild Type
ift-140(syb1325)V.(P702A);[ift-74(cas499[ift-74::gfp])]
Wild Type
syb1325
ift-140(syb1325)V.(P702A);[kyIs141[osm-9::GFP5 + lin-15(+)]]
Wild Type
ift-140(syb1325)V.(P702A);[CHE-11::GFP +pRF4]
Wild Type
syb1325
Wild Type
syb1325
