# Supplementary material for: Matching variants for functional characterization of genetic variants
Source: G3 (Bethesda). 2023 Nov 2;13(12):jkad227. doi: 10.1093/g3journal/jkad227 (PMC10700107; doi:10.1093/g3journal/jkad227)
Supplement: jkad227_Supplementary_Data [file jkad227_supplementary_data.zip › Supplementary_Table_2-3_G3-2023-404469.docx]

**Supplementary Figure 1:** Shown are PCR gel electrophoresis results for confirmation of *ift-140(P702A)* mutants with the indicated transgenic strains. Primers used for confirmation PCRs were provided in Supplementary Table 4. Wild type does not show a band, but the syb1325 mutant show a band.

**Supplementary Table 2 sgRNA target sites used in this study**

|  | **Gene locus** | **SgRNA target sites** | **Sequence information (5’→3’)** |
| --- | --- | --- | --- |
| 1 | IFT-140(G680S) | 1 | GCATTTGTAACTTCTGAACAT**GG** |
|  |  | 2 | GAACAT**GGA**ATACAACTTCAAGG |
| 1 | IFT-140(P702A) | 1 | GAACATGGAATACAACTTCAAGG |
|  |  | 2 | TTTTGTTCGGAAATCTGGATGGG |

**Supplementary Table 3 Molecular information of each CRISPR mutants generated**

|  | **Site** | **Sequence** |
| --- | --- | --- |
| 1 | N2 | GTTTTAACAGCATTTGTAACTTCTGAA**CATGGA**ATACAACTTCAAGGAATGCAGCAAAAAAAT |
|  | G680S | GTTTTAACAGCATTTGTAACTTCTGAA**CACTCG**ATACAACTTCAAGGAATGCAGCAAAAAAAT |
| 2 | N2 | **CAACTT**CAAGGAATGCAGCAAAAAAATCTGCATTGTGGAAAACTTGTTTCAGTCTCT**GTTCCA**AATTTTTATTTTGTTCGGAAATCT**GGA**TGG |
|  | P702A | **CAGCTG**CAAGGAATGCAGCAAAAAAATCTGCATTGTGGAAAACTTGTTTCAGTCTCT**GTCGCG**AATTTTTATTTTGTTCGGAAATCT**GGT**TGG |

The codon of the target amino acid is labelled in red. All synonymous mutations (in blue) are generated to make the repaired site resistant to re-cutting by Cas9/sgRNA.
