# Supplementary figures and images for: Matching variants for functional characterization of genetic variants
Source: G3 (Bethesda). 2023 Nov 2;13(12):jkad227. doi: 10.1093/g3journal/jkad227 (PMC10700107; doi:10.1093/g3journal/jkad227)

A

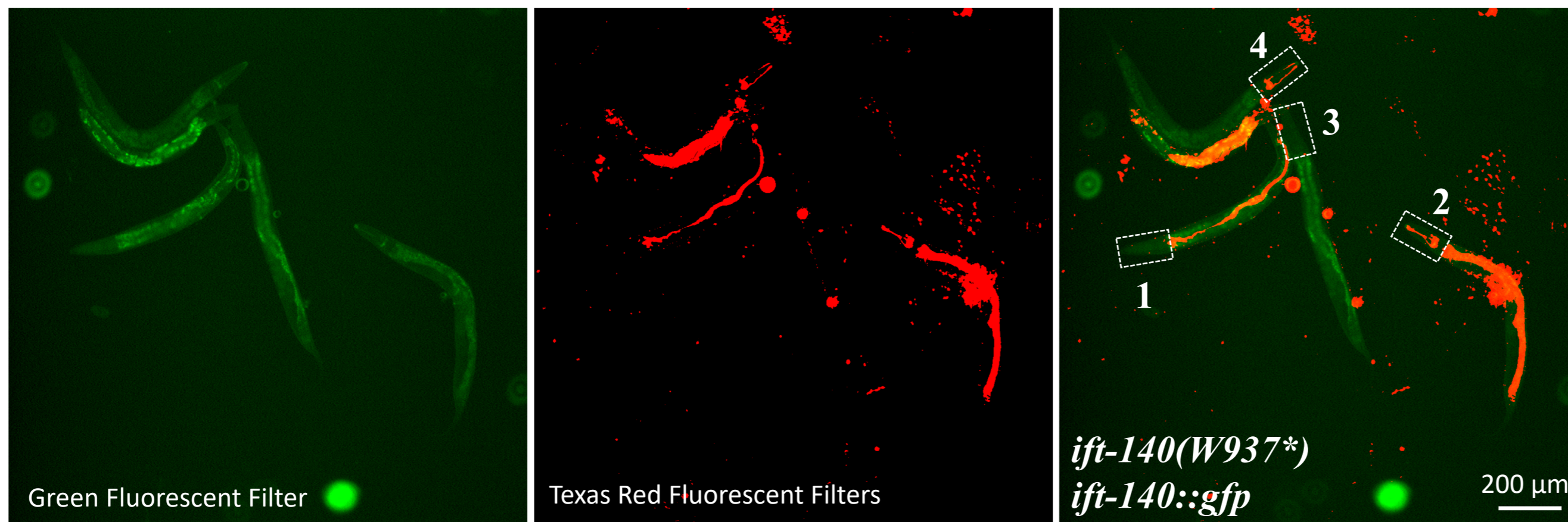

B

## AWB cilia length rescue

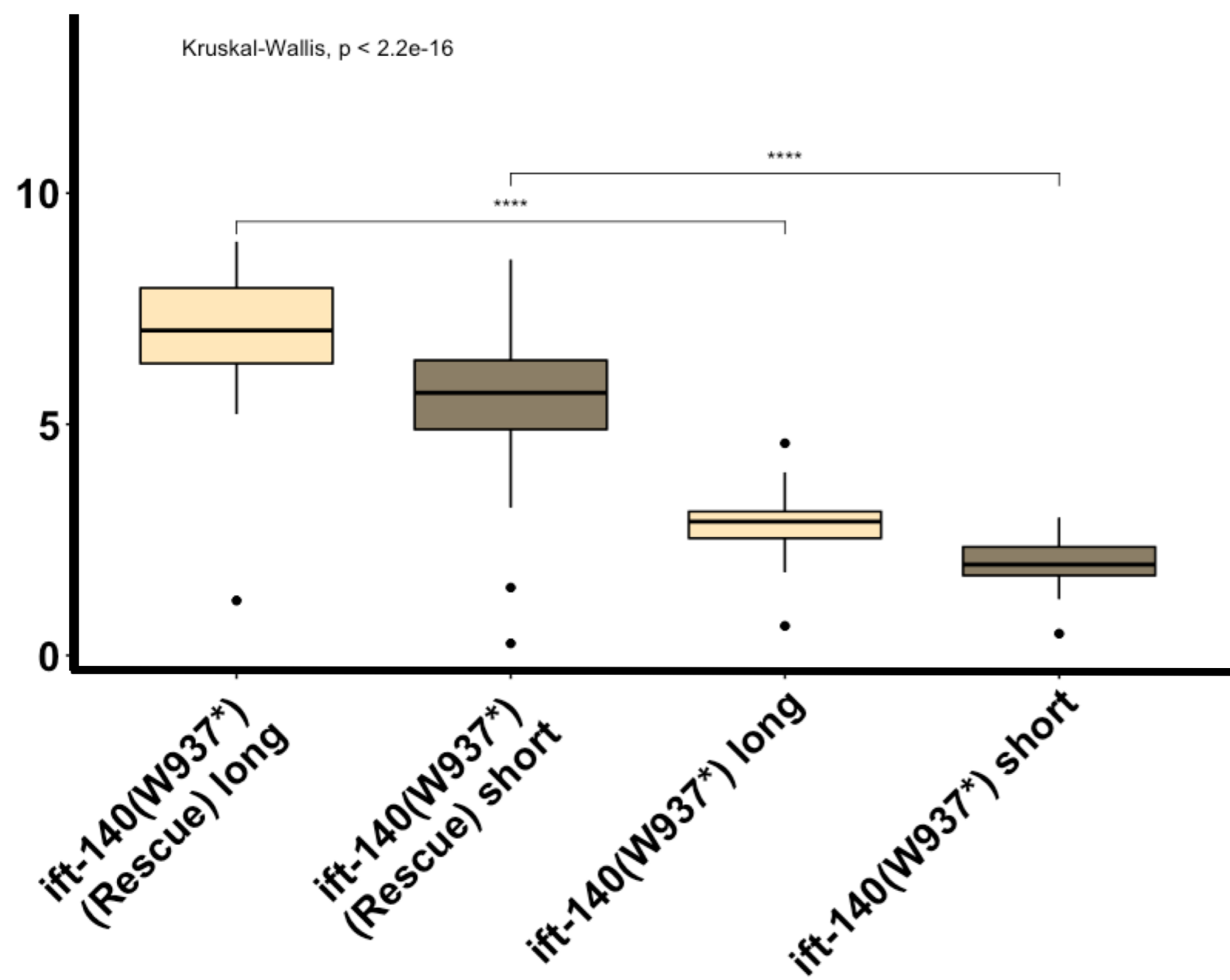

C

## AWB cilia length

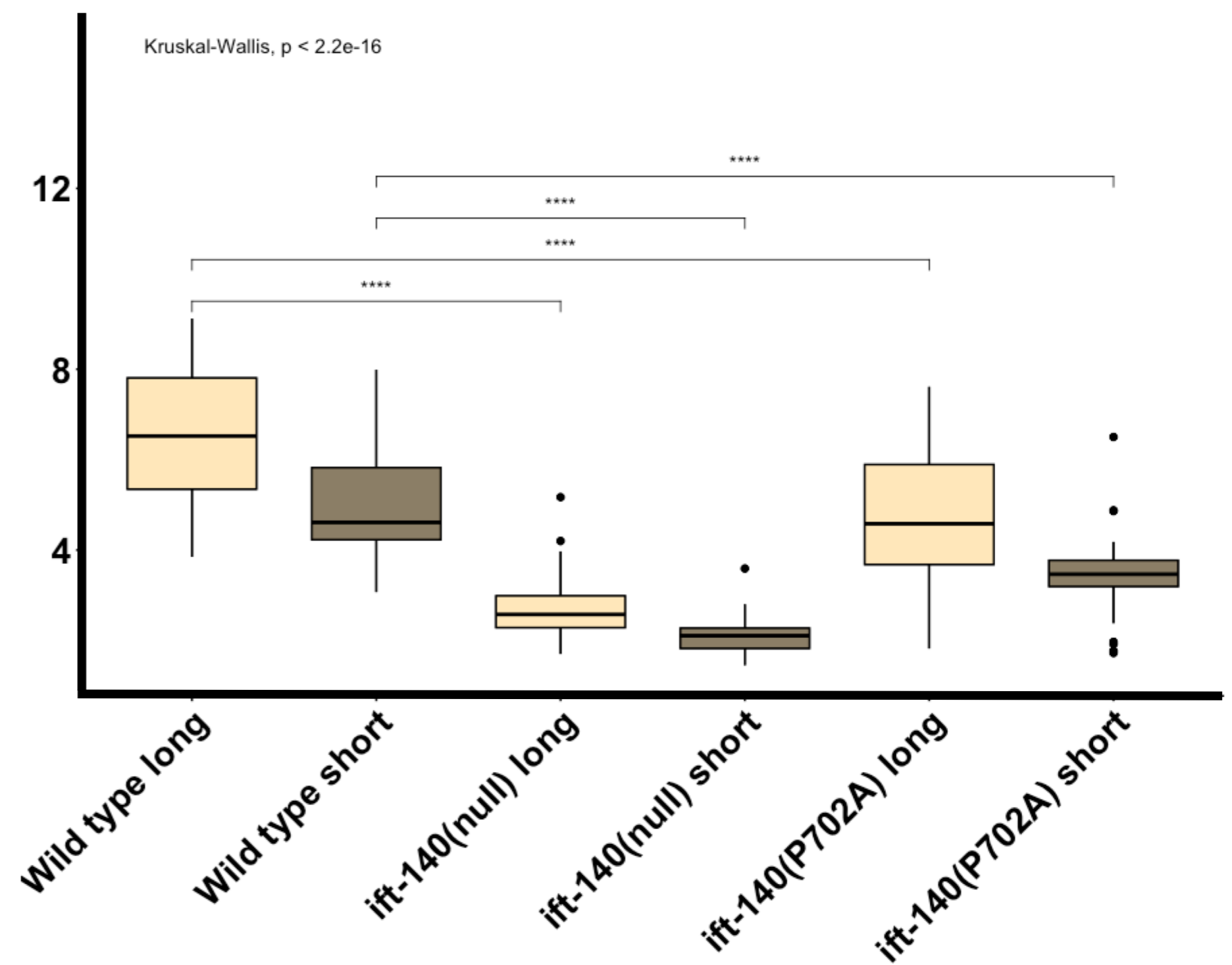

Supplement: jkad227_Supplementary_Data [file jkad227_supplementary_data.zip › Supplementary_Figure_2_G3-2023-404469.pdf]

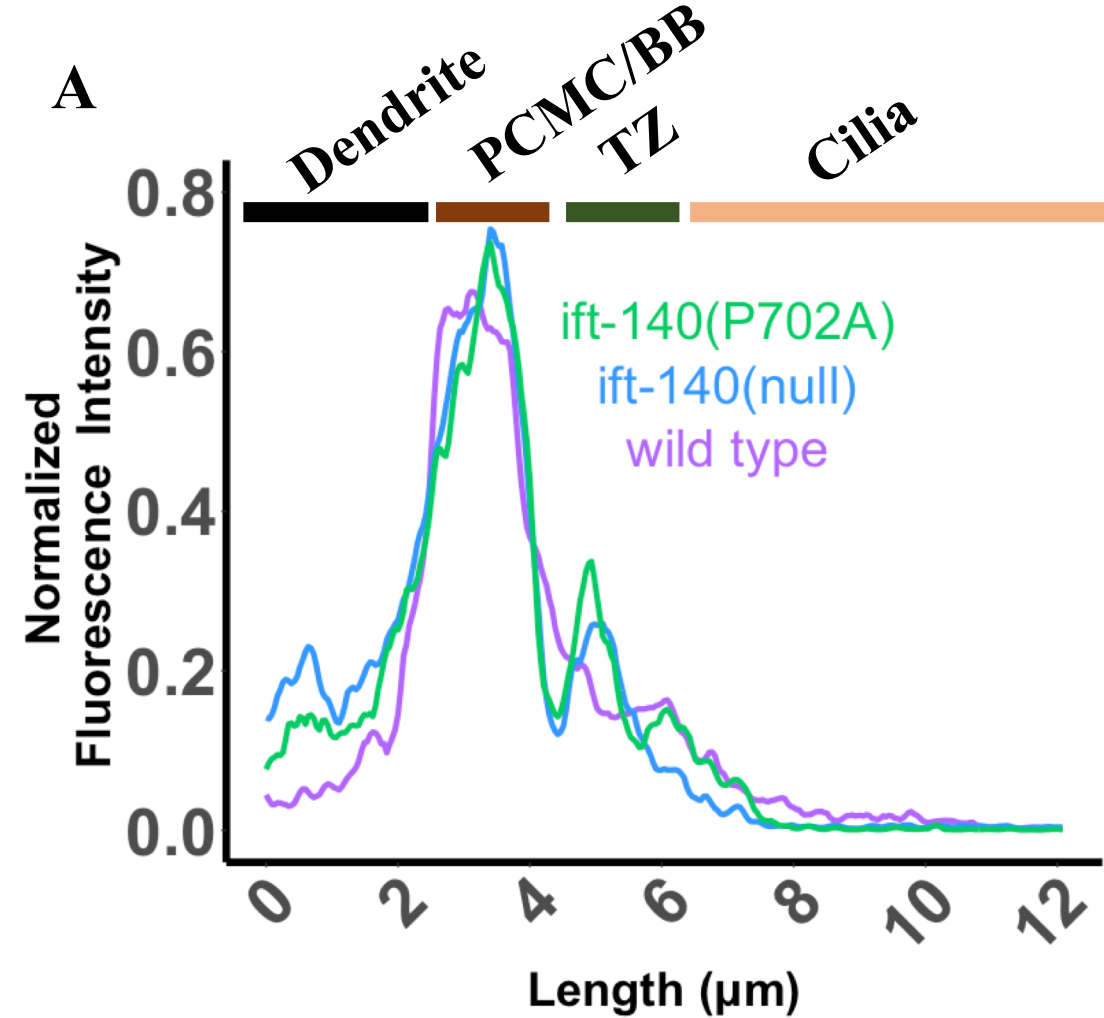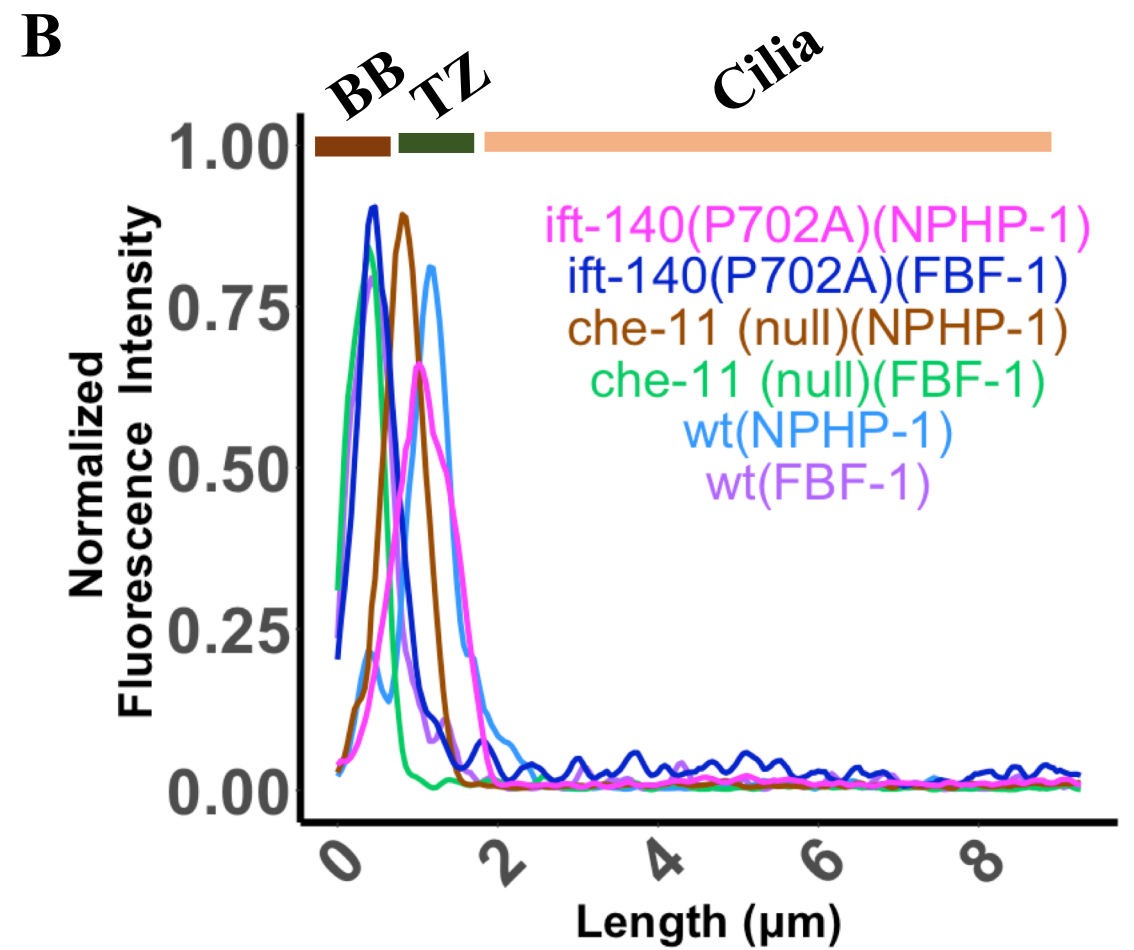

Supplement: jkad227_Supplementary_Data [file jkad227_supplementary_data.zip › Supplementary_Figure_3_G3-2023-404469.pdf]
